# Development of CFTR Structure

**DOI:** 10.3389/fphar.2012.00162

**Published:** 2012-09-06

**Authors:** Anna E. Patrick, Philip J. Thomas

**Affiliations:** ^1^Department of Physiology, University of Texas Southwestern Medical CenterDallas, TX, USA

**Keywords:** CFTR, cystic fibrosis, ABC transporter, membrane protein structure, multidomain protein folding

## Abstract

Cystic fibrosis is a lethal genetic disease caused by lack of functional cystic fibrosis transmembrane conductance regulator (CFTR) proteins at the apical surface of secretory epithelia. CFTR is a multidomain protein, containing five domains, and its functional structure is attained in a hierarchical folding process. Most CF-causing mutations in CFTR, including the most common mutation, a deletion of phenylalanine at position 508 (ΔF508), are unable to properly fold into this functional native three dimensional structure. Currently, no high-resolution structural information about full length CFTR exists. However, insight has been gained through examining homologous ABC transporter structures, molecular modeling, and high-resolution structures of individual, isolated CFTR domains. Taken together, these studies indicate that the prevalent ΔF508 mutation disrupts two essential steps during the development of the native structure: folding of the first nucleotide binding domain (NBD1) and its later association with the fourth intracellular loop (ICL4) in the second transmembrane domain (TMD2). Therapeutics to rescue ΔF508 and other mutants in CFTR can be targeted to correct defects that occur during the complex folding process. This article reviews the structural relationships between CFTR and ABC transporters and current knowledge about how CFTR attains its structure–with a focus on how this process is altered by CF-causing mutations in a manner targetable by therapeutics.

## Introduction

Cystic fibrosis (CF) is an autosomal recessive disease affecting more than 70,000 people world-wide. CF is caused by mutations in the gene encoding the CF transmembrane conductance regulator (CFTR) protein (Kerem et al., 1989; Riordan et al., 1989; Rommens et al., 1989). CFTR functions as a regulated chloride channel in the apical membrane of epithelia, where it plays a critical role in maintaining the surface liquid layer. Lack of functional CFTR results in thick secretions that cause gastrointestinal, reproductive, and respiratory system defects. Currently, CF patients most commonly die of respiratory-associated problems.

More than 70% of CF patients have at least one allele with a deletion of phenylalanine at position 508 (ΔF508; Kerem et al., 1989). Further sequencing of CF patient and non-patient CFTR genes has been extensive, and hundreds of mutations have been identified^1^. Many of these mutations have been validated as CF-causing, while others are CF-associated but unstudied. The validated CF-causing mutations are located throughout the CFTR gene, and are inherited in almost all cases (Riordan et al., 1989; Riordan, 2008). ΔF508 (Cheng et al., 1990; Thomas et al., 1992) and many other CF mutations (Gregory et al., 1991) result in mutant CFTR that does not properly fold and is retained in the ER by cell protein quality control. The result is that more than 90% of mutant CFTR alleles produce a misfolded protein that is recognized, mistrafficked, and degraded in the cell.

While we do not have high-resolution three dimensional structural information for full length CFTR, a great deal of correlative information regarding this structure has been obtained *via* homologous structures, domain structures, molecular modeling, and lower resolution techniques.

## ABC Transporters

**Figure 1:**
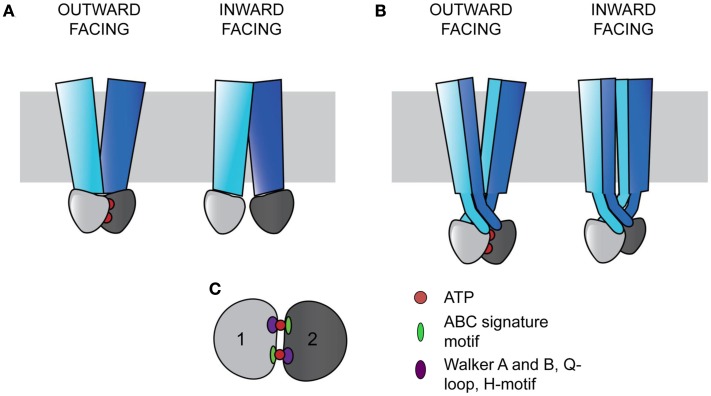
**ABC transporters contain a core architecture of two TMDs (blue and cyan) and two NBDs (light and dark gray)**. Signals from the NBDs relating to ATP binding and hydrolysis result in wing-like TMD movements that transport substrates across the membrane (gray rectangle). In the outward facing configuration, the two NBDs bind ATP (red circles) and are close together, with the TMDs open to the non-cytosolic side. In the inward facing configuration, the NBDs are more distant without ATP bound, and the TMDs are open to the cytosol. **(A)** In ABC importers, each TMD interacts with a single NBD (Examples include BtuCD and MalFGK). **(B)** In ABC exporters, the TMDs wrap around each other in a domain-swapped fashion, with each TMD interacting with both NBDs (Examples include Sav1866 and MsbA). **(C)** The two NBDs sandwich two ATPs in a head-to-tail fashion. Each ATP binding and hydrolysis site is comprised of both NBDs, containing the conserved structural motifs in the catalytic subdomain including the Walker A and B, Q-loop, and H-motif (purple oval), and the ABC signature motif in the alpha-helical subdomain (green oval).

Cystic fibrosis transmembrane conductance regulator is a member of the ATP-binding cassette (ABC) transporter superfamily of proteins, which includes membrane spanning proteins that use nucleotide hydrolysis to transport substrates across the membrane bilayer (Holland, 2003). While there is no full length high-resolution structure for CFTR, there are structures for other ABC transporters, providing insight into the structure arrangement and functional mechanisms of CFTR. Most ABC transporters function to move substrates either into the cytoplasm (importers) or out of the cytoplasm (exporters). Exporters are found in both eukaryotes and prokaryotes, while importers have only been found in prokaryotes (Rees et al., 2009). The importance of prokaryotic ABC transporters for cellular functions, such as import of nutrients and export of toxins, is highlighted by their representation as 5% of the *Escherichia coli* genome (Linton and Higgins, 1998). In humans, 48 or 49 distinct ABC transporters have been identified, many of which are implicated in disease (Dean et al., 2001; Gottesman and Ambudkar, 2001; Borst and Elferink, 2002). The core ABC transporter architecture is comprised of two transmembrane spanning domains (TMDs) and two nucleotide binding domains (NBDs). Many transporters also have accessory domains with regulatory functions (Biemans-Oldehinkel et al., 2006). In general, the TMDs are organized as two wings that open and close in response to NBD movements resulting from ATP binding and hydrolysis (Figure 1; Moody et al., 2002; Smith et al., 2002; Locher, 2009; Rees et al., 2009). Additionally, at the external surface, many prokaryotic importers interact with accessory proteins that play a role in substrate transport (Biemans-Oldehinkel et al., 2006). The domains are modular, and are found expressed individually, in combinations, or as a single full length transporter to form the functional protein (Locher, 2009).

ATP-binding cassette transporters have a conserved coupling mechanism, whereby signals from the NBDs are transmitted to the intracellular loops (ICLs) of TMDs to cause substrate transport (Locher, 2009). The conserved NBDs form a sandwich around two ATPs, with each site for ATP binding and hydrolysis requiring both domains (Smith et al., 2002). Two subdomains are present in each NBD. The catalytic subdomain contains the conserved Walker A and B motifs, a Q-loop, and an H-motif, and the alpha-helical subdomain contains the ABC signature motif, LSGGQ (Figure 1C; Rees et al., 2009). Each active site is composed of components from the catalytic subunit of one NBD and the alpha-helical components of the other NBD in a head-to-tail arrangement (Smith et al., 2002; Rees et al., 2009). The binding of ATP in these sites drives the association of the NBDs (Moody et al., 2002).

The TMDs are proposed to function in an alternating access model of transport and are the most variable among ABC transporters (Chen et al., 2001; Dawson et al., 2007). ABC transporters can be divided into three classes based on the TMD fold (Locher, 2009). Type I and II ABC importers contain different core transmembrane (TM) span topologies of 10 and 20 TM helices respectively, with the latter tending to facilitate transport of larger substrates (Locher et al., 2002; Hollenstein et al., 2007; Locher, 2009). In both importer types, one TMD interacts with one NBD to form two TMD-NBD units that together form a functional transporter (Figure 1A; Locher, 2009). ABC exporters contain a core of 12 TM helices, with each wing of the transporter made of both TMDs, with each TMD interacting with both NBDs in a domain-swapped fashion (Figure 1B; Dawson and Locher, 2006; Locher, 2009). In this arrangement, the ICLs extend into the cytoplasm, positioning the NBDs approximately 25 Å from the membrane (Figure 1B; Locher, 2009). In exporters, the TMDs and NBDs are expressed as TMD-NBD units, and eukaryotic exporters are most frequently found as full length transporters (Nikles and Tampe, 2007).

## CFTR as an ABC Transporter

**Figure 2:**
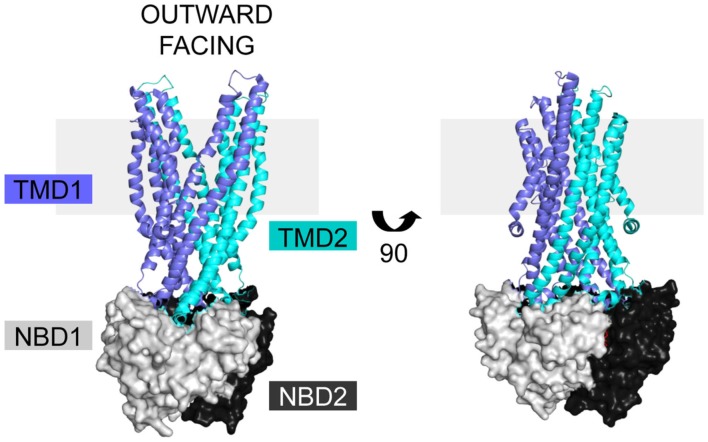
**CFTR homology models are based on the ABC exporter structures**. The Sav1866 (pdb 2HYD) exporter is shown with domains colored based on representative CFTR domains, with TMD1 (blue), NBD1 (light gray), TMD2 (cyan), and NBD2 (black). There is no homologous structure for the CFTR R domain. The structure is in the outward facing configuration, which for CFTR is the open channel. The view is shown in the plane of the membrane (gray rectangle).

Cystic fibrosis transmembrane conductance regulator is a member of the ABC C subfamily, and is structurally homologous to the domain-swapped exporters. Structures of homologous ABC exporters such as bacterial Sav1866 (Dawson and Locher, 2006, 2007), bacterial MsbA (Ward et al., 2007), bacterial TM287/288 (Hohl et al., 2012), and mammalian P-glycoprotein (Aller et al., 2009) have been solved. The available structural data in combination with sequence alignments form the basis for homology models of full length CFTR that provide insight into its structure, mechanisms of regulation, and signal transduction (Mendoza and Thomas, 2007; Mornon et al., 2008, 2009; Serohijos et al., 2008). The exporter structures are in both open and closed forms, giving insight into movements within the CFTR protein during a transport cycle (Figure 2, open form; Ward et al., 2007; Locher, 2009; Mornon et al., 2009; Rees et al., 2009). The similarity of CFTR movements to other ABC transporters is supported by electron microscopy data in combination with a low resolution crystal structure (Rosenberg et al., 2004, 2011; Zhang et al., 2009, 2011). The only high-resolution structures of CFTR domains are of NBD1 (Lewis et al., 2004, 2005, 2010; Thibodeau et al., 2005) and NBD2 (pdb 3GD7). As an ABC transporter, CFTR contains two TMDs, two NBDs, and a unique regulatory R region translated from an mRNA transcript as a single polypeptide chain (Riordan et al., 1989). Sav1866 based CFTR models have extensive interdomain interactions between the TMDs and NBDs, but lack regions without sequence homology, like the R domain (Figure 2; Dawson and Locher, 2006; Mendoza and Thomas, 2007).

The NBDs of CFTR, like other ABC transporters (Moody et al., 2002; Smith et al., 2002), interact in a head-to-tail fashion forming two sandwiched ATP binding pockets made of both domains (Vergani et al., 2005; Mense et al., 2006). Each NBD has a catalytic subdomain that contains the Walker A and B motifs and an alpha-helical subdomain that contains the conserved ABC signature motif (Lewis et al., 2004; Thibodeau et al., 2005). However, like several other members of the ABC C subfamily, one ATP binding site is non-hydrolytic (Muallem and Vergani, 2009). In this site, non-conservative mutations, which are located in the NBD1 Walker B and switch motifs and in the NBD2 signature sequence, result in tight binding and inefficient ATP hydrolysis (Aleksandrov et al., 2002; Basso et al., 2003; Gadsby et al., 2006). In general, CFTR ATP driven conformational changes include ATP binding, which results in an NBD dimer that signals the TMDs to open. Then, hydrolysis of one ATP disrupts the NBD interface, the NBDs separate, and the channel closes (Gadsby et al., 2006; Aleksandrov et al., 2007; Muallem and Vergani, 2009). However, the driving forces that control the gating transitions and the signals transmitted by ATP binding and hydrolysis are a matter of debate (Gadsby et al., 2006; Aleksandrov et al., 2007; Muallem and Vergani, 2009). NBD1 also contains two non-conserved regions, a regulatory insert (RI) near the N-terminus and a regulatory extension (RE) near the C-terminus. Of these two regions, studies have focused on the RI. The RI is disordered in the NBD1 crystal structures and plays a role in regulation of CFTR channel gating, but is not required for trafficking in the cell (Lewis et al., 2004; Thibodeau et al., 2005; Aleksandrov et al., 2010). Furthermore, a mechanism wherein RI movements alter ICL1-NBD1 interactions to affect phosphorylation-dependent CFTR gating has been proposed (Kanelis et al., 2010).

Like other domain-swapped exporters, the TMDs form two wings containing TMs from both TMD1 and TMD2, such that the first two TMs and last four TMs of each domain make a wing (Figure 2; Dawson and Locher, 2006; Mendoza and Thomas, 2007). Based on the exporter structures, the wings move to open and close the chloride channel for ion transport (Vergani et al., 2005; Mornon et al., 2009). In each TMD, two alpha-helical ICLs extend into the cytoplasm, with each having a distal coupling helix that interacts with the NBDs (Mendoza and Thomas, 2007; Mornon et al., 2008; Serohijos et al., 2008). In combination, the four ICLs form four helix inner and outer bundles that end in the coupling helices (Figure 2; Mornon et al., 2008, 2009). Each coupling helix is parallel to the NBD surface, and forms a largely hydrophobic interface (Mendoza and Thomas, 2007). In CFTR, ICL2 interacts with NBD2, ICL4 interacts with NBD1, and ICLs 1 and 3 interact with both NBD1 and NBD2. Importantly, the F508 position in NBD1 is predicted to lie near the interface between NBD1 and ICL4 (Mendoza and Thomas, 2007). Many of the predicted interdomain interactions are also experimentally validated by crosslinking studies (Chen et al., 2004; Mense et al., 2006; He et al., 2008; Loo and Clarke, 2008; Serohijos et al., 2008). Further complexity of the ICL-NBD interactions is generated by phosphorylation-dependent interactions between NBD1 and an ICL1 peptide (Kanelis et al., 2010). Additionally, crosslinks between an ICL and the opposing NBD disrupt channel opening, supporting the essential roles of these components for channel function (He et al., 2008). Models predict specific residues are critical for the interactions between the ICLs and NBDs, including Y275 and W277 which form an interface with NBD2 (He et al., 2008; Mornon et al., 2008); D173, S169, and R170 which are predicted to contact nucleotide and NBD1 (Mornon et al., 2008); and S263 and E267 which stabilize ICL helical bundle structure (Mornon et al., 2009). Notably, the W277 position is equivalent to the R1070 position in ICL4 (Mornon et al., 2008) that when mutated, R1070W, suppresses the ΔF508 mutation (Thibodeau et al., 2010; Mendoza et al., 2012). These positions have not yet been fully tested for their roles in the folding and function of CFTR.

In summary, conformational signals generated in the NBDs in relation to ATP binding and hydrolysis are transmitted by the ICLs in the TMDs, resulting in chloride channel opening and closing (Gadsby et al., 2006; Riordan, 2008). The interactions between CFTR ICLs and NBDs have been validated by crosslinking studies (He et al., 2008; Serohijos et al., 2008) and complementation of a mutant located in an NBD with a mutant in an ICL (Thibodeau et al., 2010). The coupling helices of ABC transporters are architecturally conserved without having a highly conserved sequence (Locher, 2009), making prediction of essential positions and residues difficult without a high-resolution full length CFTR structure.

Cystic fibrosis transmembrane conductance regulator is the only known channel among the ABC transporters. In the alternating access model, ABC transporters are open to one side of the membrane bilayer at a time (Chen et al., 2001; Dawson et al., 2007). In CFTR, channel formation abrogates this model, as one of the gates that would normally block substrate transport must be atrophied or gone to allow chloride flux (Gadsby, 2009). With regard to this, CFTR has been called a broken ABC transporter (Jordan et al., 2008; Muallem and Vergani, 2009). Similar to other chloride channels, CFTR is not very selective among small monovalent anions and has a relatively featureless pore (Gadsby et al., 2006; Gadsby, 2009). Putative residues that make the chloride channel have been identified in TMs and in extracellular loops, with a focus on TM1 and TM6 (Linsdell, 2006). However, it is difficult to validate these residues without better characterizing the TM span positions and TMD structures. Further complicating the TMD structure is a TMD1 N-terminal cytosolic region that regulates CFTR channel activity through interactions with the R domain, neither of which has a homologous structure (Naren et al., 1999; Chappe et al., 2005).

The chloride channel activity of CFTR is regulated by the R domain (Riordan, 2008). The R domain is largely unstructured and has multiple sites that are phosphorylated by PKA, resulting in CFTR channel activation (Gadsby et al., 2006; Baker et al., 2007). Consistent with this, the unphosphorylated R domain has an inhibitory effect on the CFTR channel (Rich et al., 1991; Csanady et al., 2000). The R domain interacts with multiple other regions of CFTR, including NBD1 and the N-terminus of TMD1 (Naren et al., 1999; Baker et al., 2007; Kanelis et al., 2010). This evidence suggests the R domain may act as a signal integrator to regulate channel function via interactions with different regions of CFTR. However, due to its lack of homology and disordered nature, the R domain location within CFTR models remains unclear.

Many different modifications to the CFTR protein that may impact its structure have been identified. For instance, CFTR contains two *N*-linked glycosylation sites, NXS/T (X≠P), within TMD2 that are core glycosylated in the ER lumen. This core glycosylation is then modified in the Golgi to produce complex glycosylated protein (Helenius and Aebi, 2001). The natural sites within CFTR are regularly used to monitor its integration and cellular trafficking by changes in electrophoretic mobility upon core glycosylation, producing Band B at approximately 150 kDa, and complex glycosylation, producing a diffuse Band C above 170 kDa. The natural glycosylation sites are not required for cellular trafficking from the ER and chloride channel function (Howard et al., 1995; Chang et al., 2008; Glozman et al., 2009; Patrick et al., 2011). However, recently, these sites have been found to influence the efficiency of CFTR productive protein folding and early secretory trafficking (Glozman et al., 2009), and cell surface retention and turnover in post-ER cellular compartments (Chang et al., 2008; Glozman et al., 2009). The impact of these and other modifications on the development of CFTR structure is an area of ongoing study.

The combination of experimental and modeling studies provides significant insight into the CFTR structure, which allows formation of models within which mechanochemical mechanisms and the effect of CF-causing folding mutations can be framed. However, since many CF-causing mutations, including ΔF508, result in misfolding of the CFTR protein, the folded full length structure may not adequately describe the relevant defects.

## CFTR Folding as a Multidomain Protein

**Figure 3:**
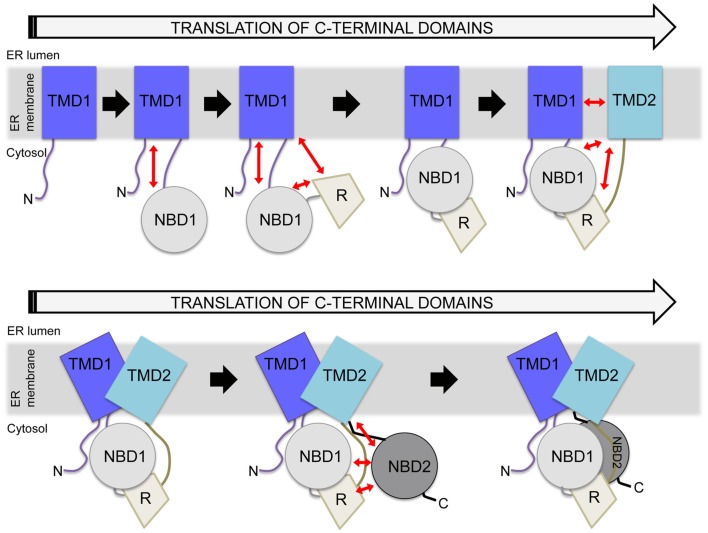
**Hierarchical folding model of CFTR**. Potential interdomain interactions are indicated by red arrows, with several possible structural units included during the translation process. In the cell, constructs lacking TMD2 do not traffic from the ER, whereas constructs containing TMD2 can traffic from the ER. Reflecting this, a major structural rearrangement is depicted in the presence of TMD2, as shown in the bottom panel. Eventually, NBD2 is incorporated as the final step. There are many points during this process at which the cell may monitor perturbations.

Cystic fibrosis transmembrane conductance regulator, like other ABC transporters, contains extensive interdomain surfaces (Rees et al., 2009) that, in the case of CFTR, likely form during translation (Zhang et al., 1998; Du et al., 2005; Kleizen et al., 2005; Thibodeau et al., 2005). During protein translation, secondary structure can begin to form early, even while the nascent chain is in the tunnel of the ribosome (Kramer et al., 2001; Woolhead et al., 2004). For CFTR, as translation continues each domain folds and can then interact with previously translated domains to form multidomain folding intermediates (Figure 3; Lukacs et al., 1994; Du et al., 2005; Kleizen et al., 2005; Thibodeau et al., 2005; Cui et al., 2007; Cheung and Deber, 2008; Du and Lukacs, 2009). The current model of CFTR folding holds that individual domain structures form cotranslationally (Kleizen et al., 2005). Then, intermediate structures form and eventually a TMD1-NBD1-R-TMD2 structure is produced that is required for cellular trafficking (Meacham et al., 1999; Du et al., 2005; Cui et al., 2007; Du and Lukacs, 2009). Finally, NBD2 posttranslationally incorporates into the CFTR structure (Figure 3; Du et al., 2005). The addition of NBD2 confers a greater folding efficiency and trafficking from the ER. Thus, although NBD2 is not strictly required for CFTR trafficking (Pollet et al., 2000; Cui et al., 2007; Du and Lukacs, 2009; Thibodeau et al., 2010), its posttranslational association into the CFTR structure (Du et al., 2005) may increase the yield of folded cellular CFTR. Much of this model is based on individual CFTR domains forming protease-resistant structures during translation (Zhang et al., 1998; Kleizen et al., 2005). The order of interdomain interaction formation and whether initial interactions are the same as those in the final CFTR structure is not known.

Cystic fibrosis transmembrane conductance regulator folding occurs during translation as a linear polypeptide (Riordan et al., 1989). However, many ABC transporter domains are expressed separately, and later associate to form a functional transporter (Locher, 2009). To some extent, CFTR retains some ability to fold in this manner. CFTR can be expressed as a split construct, which forms structure that traffics to the cell surface and functions as a chloride channel (Ostedgaard et al., 1997; Chan et al., 2000; Csanady et al., 2000; Du and Lukacs, 2009). Additionally, expression of constructs containing TMD1-NBD1-R or R-TMD2-NBD2 formed chloride channels, likely as multimers (Sheppard et al., 1994; Devidas et al., 1998). Finally, in the cell the minimal construct that traffics from the ER contains TMD1-NBD1-R-TMD2, which forms a chloride channel (Cui et al., 2007). These studies suggest that the domains of CFTR, to a certain extent, can associate posttranslationally to form a functional chloride channel. Yet, CFTR is a linear chain, such that folding requires that each domain attain structure in a more spatially confined manner. The critical role of the primary sequence in the CFTR folding process is highlighted by the multitude of CF-associated folding mutations identified throughout the protein (see text footnote 1).

## CFTR Cotranslational Folding Involves Interactions with Other Proteins

Cystic fibrosis transmembrane conductance regulator folding involves many proteins that act at different stages to aid folding or recognize misfolding. This topic is extensively reviewed elsewhere within this Research Topic. Briefly, the misfolded CFTR is retained in the ER, and eventually degraded (Lukacs et al., 1994) by the proteasome (Jensen et al., 1995; Ward et al., 1995). Many proteins interact with CFTR in the ER lumen, ER membrane, and cytoplasm, suggesting that the domains of CFTR are differentially monitored during the biosynthetic process. Among these identified interacting partners are the cytoplasmic proteins Hsc/p 40, 70, 90, and associated co-chaperones CHIP (Strickland et al., 1997; Meacham et al., 1999, 2001; Younger et al., 2006) and Aha1 (Wang et al., 2006), the ER membrane associated protein RMA1 (Younger et al., 2006; Grove et al., 2011), the ER integral membrane proteins Derlin (Sun et al., 2006; Younger et al., 2006; Wang et al., 2008) and BAP31 (Wang et al., 2008), and the ER luminal-interacting protein calnexin (Pind et al., 1994). After trafficking to the cell surface, CFTR interactions with cytoskeletal proteins are important for its maintenance at this cellular location (Okiyoneda and Lukacs, 2007). Also at the plasma membrane, peripheral protein quality control is involved in the ubiquitination, internalization, and degradation of misfolded CFTR (Okiyoneda et al., 2010). Moreover, a protein interactome for CFTR includes potential interactions far beyond those that have been studied (Wang et al., 2006). However, it is not clear which proteins interact at the earliest stages of folding/maturation and are responsible for initial and irreversible recognition of mutant CFTR. Furthermore, the structural aspects of CFTR during folding that are important for formation of these interactions are unclear. These interactions paint a picture of CFTR biogenesis whereby normal structural formation and interactions are formed with cellular folding and quality control machinery, providing multiple points to monitor CFTR folding.

## CF-Mutants Perturb CFTR Cotranslational Folding

Cystic fibrosis-associated mutations have been found in every domain of CFTR (see text footnote 1). Misfolded CFTR, specifically the ΔF508 mutant protein, is recognized by cellular quality control machinery, accumulates in the ER (Cheng et al., 1990), and is eventually degraded (Lukacs et al., 1994) by the proteasome (Jensen et al., 1995; Ward et al., 1995). Many studies have identified CF-causing mutants that result in accumulation of CFTR in the ER. Mutant effects have been categorized into classes based on the resulting effect on CFTR (Welsh and Smith, 1993; Zielenski and Tsui, 1995). The alterations include lack of protein production (class I), defective protein maturation and early degradation (class II), defective regulation of ATP interactions (class III), reduced chloride transport (class IV), reduced transcripts though splicing or promoter defects (class V), and increased cell surface turnover (class VI; Welsh and Smith, 1993; Zielenski and Tsui, 1995). The ΔF508 mutation accounts for 70% of CF-causing mutant CFTR alleles (Riordan et al., 1989), making class II defects the most common cause of CF.

Mutations within the CFTR protein, including ΔF508, may perturb local protein structure and/or domain structure, or could be surface exposed and perturb interactions with other domains or proteins. For instance, in the NBD-ICL4 interface, mutants in ICL4 including L1065P, R1066C, and A1067T alter trafficking and chloride channel function (Cotten et al., 1996; Seibert et al., 1996). Mutants in different domains alter biogenic intermediates of CFTR, suggesting that misfolding does not require full length CFTR (Du and Lukacs, 2009). Furthermore, in full length CFTR, the proteolytic stability of all domains was reduced for spatially separate mutations, suggesting propagation of one mutant to other domains (Rosser et al., 2008; Du and Lukacs, 2009). The propagation of mutants could occur through a rearrangement step involving multiple domains (Du and Lukacs, 2009), or through coupled folding of the domains. As discussed, various components of cell quality control recognize CFTR as it is created, such that domain and multidomain states are likely differentially monitored (Younger et al., 2006). For each mutation, the effect on individual domain folding and multidomain units plays a fundamental role in determining the mechanisms by which that mutation is recognized and managed within the cell.

An example of mutants similarly located within CFTR with different local mechanisms of misfolding are the G85E and G91R mutations. These mutations are located near or within the TM1 span within TMD1. Both mutations have been demonstrated to disrupt later steps in CFTR folding, including interdomain interactions, which have been proposed to result in mutant recognition by ER quality control machinery (Xiong et al., 1997). Recently, G85E was found to dramatically alter the conformation/integration profile of TM1 (Patrick et al., 2011). Such an alteration would occur at the earliest steps of translation and integration, and could be recognized as a very early misfolding event by ER quality control machinery. The G91R mutant was predicted to have a similar effect on CFTR (Xiong et al., 1997), but this proved not to be true with regards to the TM1 conformation/integration profile (Patrick et al., 2011). Interestingly, the corrector compound four rescues G91R but not G85E-CFTR (Grove et al., 2009), suggesting the differences in the mutant molecular pathologies may be relevant for their ability to benefit from specific treatments to rescue defective CFTR. The detailed mechanistic study of CF-causing mutations provides a better fundamental understanding of membrane protein misfolding and mechanisms for approaching mutant specific therapy for CF patients.

## Folding of NBD1 and ΔF508-NBD1

The best studied disease-causing mutation, ΔF508, alters multiple steps during CFTR folding. Particular focus has been given to folding of NBD1, wherein F508 resides. High-resolution crystal structures of both NBD1 and ΔF508-NBD1 have been solved (Lewis et al., 2004, 2005; Thibodeau et al., 2005; Mendoza et al., 2012). These structures place F508 on the domain surface, and ΔF508 does not cause significant perturbations in the crystal structure (Lewis et al., 2005). However, ΔF508-NBD1 has an increased tendency to aggregate and is destabilized, indicating a disruption during folding that is not represented in these native structures (Qu and Thomas, 1996; Lewis et al., 2005; Thibodeau et al., 2005). Consistent with this, a non-native conformation of NBD1 has been identified that is promoted by ΔF508 and linked to increased aggregation (Hoelen et al., 2010; Richardson, unpublished data). The NBD1 structure is obtained cotranslationally (Kleizen et al., 2005; Hoelen et al., 2010; Khushoo et al., 2011). During translation, a ligand-dependent N-terminal compact structure forms, and upon completion of NBD1 translation another compact structure forms (Khushoo et al., 2011). The compact N-terminal structure is not affected by ΔF508, suggesting that the folding error likely occurs at a later step of NBD1 folding (Khushoo et al., 2011). The ΔF508 misfolding begins in NBD1, making this an attractive target for correcting ΔF508-CFTR. The ΔF508 mutant effects can be partially rescued independently by suppressor mutations within NBD1 (Teem et al., 1993; Qu et al., 1997; DeCarvalho et al., 2002; Hoelen et al., 2010). Importantly, the ΔF508 effects on NBD1 also manifest during translation of the full length CFTR (Kleizen et al., 2005).

In full length CFTR, ΔF508 effects multidomain stability and interdomain interactions. In mammalian cells, the ΔF508-CFTR misfolds, resulting in cellular mistrafficking via its accumulation in the ER (Cheng et al., 1990). As shown by limited proteolysis and pulse chase analysis, the ΔF508 mutation destabilizes NBD1 and multidomain folding intermediates, implying a more global destabilization of the entire ΔF508-CFTR (Zhang et al., 1998; Meacham et al., 1999; Du et al., 2005; Cui et al., 2007; Rosser et al., 2008; Du and Lukacs, 2009). The homology model of CFTR places the F508 position at an interface between NBD1 and ICL4 of TMD2 (Mendoza and Thomas, 2007). Consistent with this, ΔF508 disrupts WT-like crosslinks between ICL4 and NBD1 and within the TMDs (Chen et al., 2004; Serohijos et al., 2008). Additionally, mutations in ICL4 can suppress the effect of ΔF508, further supporting a disruption of this interface (Thibodeau et al., 2010). Recently, the ΔF508-mediated NBD1 misfolding and multidomain assembly were both shown as essential for correction of ΔF508-CFTR (Mendoza et al., 2012; Rabeh et al., 2012). This is consistent with the known ΔF508 effects on NBD1 folding, which is a prerequisite for its interdomain interactions and formation of an NBD1 surface for ICL4 interactions. However, these experiments have not yet been able to identify the timing or mechanism(s) of domain interaction disruption. The point at which ΔF508 effects are detectable and the ability to target multiple steps to rescue the ΔF508 protein emphasizes the multistep misfolding of ΔF508-CFTR. Further details regarding this misfolding are needed to continue to rationally devise new therapeutic interventions.

Other methods to rescue ΔF508-CFTR continue to be explored. For instance, compounds have been identified that rescue ΔF508-CFTR mutation via interactions with the TMDs (Loo et al., 2011). ΔF508 and other mutant CFTRs were also partially rescued by transcomplementation, in which co-expression of parts of CFTR were able to improve trafficking of CF-mutant CFTR from the ER (Cormet-Boyaka et al., 2004; Cebotaru et al., 2008). Insights into the rescue of ΔF508-CFTR also come from the yeast homologous ABC exporter, Yor1p (Pagant et al., 2007, 2008). When a ΔF508 mimic is introduced into Yor1p, consequent mistrafficking and degradation occurs (Pagant et al., 2007). Two Yor1p suppressor mutations in the TM-ICL juncture were found to correct the ΔF508 mimic (Pagant et al., 2010), suggesting that modification of the ICL structures rather than direct stabilization of the NBD-ICL interface is a potential target for correction of ΔF508-CFTR. Also, a co-expressed Yor1p NBD1 was able to swap into the ΔF508 mimic-Yor1p to replace the defective domain (Louie et al., 2010). Notable differences exist between the Yor1p protein and CFTR; however these findings provide insight into potential mechanisms for ΔF508-CFTR correction that should be investigated directly with CFTR.

## Rescuing Mutant CFTR

It is suggested that only 10–35% of CFTR function is needed to positively impact pulmonary disease (Kerem, 2004), therefore the production and residual activity of mutant CFTR is relevant for clinical outcomes. In CF, there is a focus on rescuing the defective CFTR protein. Ongoing therapeutic developments are aimed at targeting mutations that introduce premature termination codons, decrease chloride channel function, and alter cellular trafficking, which are discussed elsewhere within this Research Topic. For ΔF508 and other missense mutations, two aspects to rescuing mutant CFTR protein are to rescue processing and function, both of which are innately linked to CFTR structure.

Thus far, great success has occurred in rescuing the CF-causing G551D mutant. G551D-CFTR has normal cell surface expression and half-life, but confers a severe defect in channel gating (Welsh and Smith, 1993). The compound VX-770 was initially characterized as a CFTR potentiator in CF airway epithelial cells (Van Goor et al., 2009). This compound has since undergone clinical trials showing efficacy in CF patients (Accurso et al., 2010; Ramsey et al., 2011), has been approved by the FDA for treatment of G551D based CF in patients over 6 years old, and is now marketed as Kalydeco^™^. These results are promising for CF patients as adults, who already have lung scarring and dysfunction, and for children, who may be able to avoid lung dysfunction with this therapeutic. This success has generated a foundation to guide further progress in CF therapeutic development for other mutants, such as ΔF508.

ΔF508 and other mutants that cause CFTR misfolding, mistrafficking, and disrupted channel function are the largest CF therapeutic target. The ΔF508-CFTR exhibits a temperature sensitive trafficking from the ER, in which it is retained in the ER at 37°C, but partially traffics from the ER at lower temperatures (Denning et al., 1992). This imparts the idea that trafficking correction is feasible for ΔF508 if a chemical compound can mimic the temperature rescue. However, ΔF508-CFTR that is induced to fold/traffic by low temperature or chemical modifier treatments has disrupted chloride channel function (Dalemans et al., 1991) and shorter residence times at the cellular surface (Lukacs et al., 1993), indicating the native structure is not achieved. This makes approaching ΔF508-CFTR a complex problem. Recently, it was found that correction of both the ΔF508-NBD1 defect and the ΔF508-NBD1-ICL4 interaction defect are required to rescue ΔF508, consistent with at least two steps for correction of ΔF508-CFTR (Mendoza et al., 2012; Rabeh et al., 2012). ΔF508 is being targeted pharmacologically by strategies that aim to correct the trafficking defect and potentiate channel function. Currently trials of VX-809 or VX-661, to correct trafficking, and Kalydeco^™^, to potentiate channel function, are ongoing. However, development of a combination therapy is exponentially more complicated and difficult. Ideally, a single compound to both correct and potentiate mutant CFTR will be identified (Sheppard, 2011). Extensive work has gone into describing ΔF508-CFTR misfolding in order to identify the most pertinent misfolding step(s) for generating the most relevant therapeutic target.

## Discussion

Cystic fibrosis transmembrane conductance regulator structural development occurs in a complex manner (Figure 3). It requires formation of TMD1, which involves TM span interactions with the translocation machinery in the ER. Then production of two cytosolic domains occurs, first NBD1 and then R. Following this, yet another TMD must be appropriately integrated, with the protein structure completed after the production of cytosolic NBD2. In the final structure, these domains form extensive interdomain interactions, with the later interaction surfaces having no obvious interaction partners prior to formation of the final structure. For instance, during translation, the TM and ICL regions that form later interdomain interactions are present minutes prior to production of their interaction partners. These regions are very hydrophobic and are unlikely to be stable without their partner sequences or other protein interactions. While the ICL helical bundle likely forms only when both TMD1 and TMD2 are present, this has not been tested experimentally. It is not known if ICL structure formation begins in TMD1, or what happens to the coupling helices before both NBDs are present. A requirement of this structure for NBD docking onto the ICLs has not been examined. Knowledge of the timing of this structure formation and its role in TMD-NBD interactions will be required for better understanding development of ABC transporter structure. The interactions required for the formation of native CFTR structure are important for understanding CF-mutant mediated misfolding, which is a therapeutic target for correcting CF-mutant CFTR.

Experimental evidence supports that the first four domains of CFTR undergo a multidomain rearrangement, since a regulated chloride channel that can traffic to the plasma membrane is formed (Cui et al., 2007). The cell is able to monitor and determine whether the TMD2 containing construct should traffic from the ER (Cui et al., 2007; Du and Lukacs, 2009; Thibodeau et al., 2010). This suggests that, upon the translation of TMD2, the protein quality control machinery makes a distinction between folded and unfolded CFTR. A hierarchical folding model also predicts that two and three domain hierarchical interactions also form (Figure 3). Though this model is appealing, little evidence exists to support domain associations prior to the translation of TMD2. The most suggestive evidence of interdomain interactions in the first two and three domains of CFTR is the formation of a more stable three domain construct (Meacham et al., 1999; Rosser et al., 2008; Grove et al., 2009). In these studies, the interdomain interactions are implied rather than directly tested. Much of the evidence for formation of multidomain units is forced to rely on the use of modeling and perturbing mutations to detect the structural units. It is clear, however, that the most highly studied mutants, specifically ΔF508, alter domain structure in a manner recognizable by the cell (Du and Lukacs, 2009), convoluting the interpretation of multidomain complexes with domain effects. A continuing effort to analyze native and mutant CFTR and to develop assays to better study multidomain unit formation are required to continue addressing these specific issues.

It is important to consider that CFTR cotranslational interactions may be directly related to the order of domain translation. If these interactions are required sequentially for structure formation, then a linear peptide should be essential to produce folded CFTR. However, CFTR expressed as two pieces underwent cellular trafficking as monitored by glycosylation (Ostedgaard et al., 1997; Chan et al., 2000; Csanady et al., 2000; Du and Lukacs, 2009), inconsistent with the model. By contrast, the ability of split CFTR to form functional protein is consistent with other ABC transporters within which the modular formation of domain structure indicates that one domain is not required for the formation of other domains (Locher, 2009). Yet, during *in vitro* refolding of the modular ABC transporter, BtuCD, refolding from partially unfolded units resulted in the highest functional measures (Di Bartolo et al., 2011). This suggests that domain interactions during folding may play a role in increasing the production yield of functional protein. For CFTR, these interactions could be potentiated by the linear arrangement and be important for generating enough functional protein to maintain normal physiology. This may play a role in reaching a level of physiologically functional CFTR required to alter the progression of CF therapeutically.

Cystic fibrosis clinically impacts multiple organ systems, such that treatment of the basic defect in CFTR is the best way to address the widespread morbidities. Novel therapeutics show tremendous promise for altering the molecular pathologies of CF, however, implementation of therapeutics designed to correct the most common mutant, ΔF508, is difficult. The ΔF508 molecular pathology is complex and involves multiple levels of misfolding and recognition thereof in the cell. Indeed, ΔF508-CFTR misfolds and is accumulated in the ER (Cheng et al., 1990). Moreover, if the trafficking defect is overcome, cell surface ΔF508-CFTR displays reduced chloride transport (Dalemans et al., 1991) and an accelerated turnover rate (Lukacs et al., 1993). Addressing each effect individually is inadequate, and a successful combination of therapeutics has not yet been identified to effectively rescue the ΔF508 mutation and remains an untraveled therapeutic path. Suppressor mutations of ΔF508 have been identified within NBD1 (Teem et al., 1993) and within ICL4 (Thibodeau et al., 2010), which correct NBD1 folding and/or multidomain folding. But, individually, these suppressors have limited efficacy. It is now established that correction of at least two steps are needed to rescue ΔF508, including NBD1 folding and interdomain interactions (Mendoza et al., 2012; Rabeh et al., 2012). If the effects of the different suppressor mutations for ΔF508 either within NBD1 or distant in the CFTR protein can be mimicked and combined in a small molecule this could prove an effective therapeutic. It is clear from these studies that the identification of disease mechanisms that may be targeted therapeutically requires a global understanding of CFTR structure. Future disease modifying compounds will be more effective if the target is the most relevant biological defect.

## Conflict of Interest Statement

The authors declare that the research was conducted in the absence of any commercial or financial relationships that could be construed as a potential conflict of interest.

## Footnotes

^1^www.genet.sickkids.on.ca
